# Zoonotic *Bartonella* species in Eurasian wolves and other free‐ranging wild mammals from Italy

**DOI:** 10.1111/zph.12827

**Published:** 2021-03-29

**Authors:** Grazia Greco, Aya Attia Koraney Zarea, Giovanni Sgroi, Maria Tempesta, Nicola D’Alessio, Gianvito Lanave, Marcos Antônio Bezerra‐Santos, Roberta Iatta, Vincenzo Veneziano, Domenico Otranto, Bruno Chomel

**Affiliations:** ^1^ Veterinary Medicine Department University of Bari Aldo Moro Valenzano Bari 70010 Italy; ^2^ Department of Microbiology and Immunology National Research Centre Cairo Egypt; ^3^ Department of Veterinary Medicine and Animal Productions University of Napoli Federico II Napoli Italy; ^4^ Istituto Zooprofilattico Sperimentale del Mezzogiorno Portici Italy; ^5^ Department of Population Health and Reproduction, School of Veterinary Medicine University of California Davis CA 95616 USA

**Keywords:** *Bartonella*, Eurasian wolf, hedgehog, red fox, roe deer, zoonosis

## Abstract

Bartonellae are emerging vector‐borne pathogens infecting humans, domestic mammals and wildlife. Ninety‐seven red foxes (*Vulpes vulpes*), 8 European badgers (*Meles meles*), 6 Eurasian wolves (*Canis lupus*), 6 European hedgehogs (*Erinaceus europaeus*), 3 beech martens (*Martes foina*) and 2 roe deer (*Capreolus capreolus*) from Italian Nature Conservatory Parks were investigated for *Bartonella* infection. Several *Bartonella* species (9.84%; 95% CI: 4.55–15.12), including zoonotic ones, were molecularly detected among wolves (83.3%; 95% CI: 51–100.00), foxes (4.12%; 95% CI: 0.17–8.08), hedgehogs (33.33%; 95% CI: 0.00–71.05) and a roe deer. *Bartonella rochalimae* was the most common *Bartonella* species (i.e. in 4 foxes and 2 wolves) detected. *Candidatus* B. merieuxii and *B. vinsonii* subsp. *berkhoffii* were identified for the first time in wolves. Furthermore, *Bartonella schoenbuchensis* was identified in a roe deer and a new clone with phylogenetic proximity to *B. clarridgeiae* was detected in European hedgehogs. Zoonotic and other *Bartonella* species were significantly more frequent in Eurasian wolves (*p* < .0001), than in other free‐ranging wild mammals, representing a potential reservoir for infection in humans and domestic animals.

**Impacts**

- A high occurrence of *Bartonella* spp. was found in Eurasian wolves (83.3%) and other wildlife of southern Italy.
- Most of the *Bartonella* species from wildlife were zoonotic, posing threats for people and domestic animals at their interface
- Wildlife disease surveillance is a useful and complementary component of human and domestic animal disease surveillance.

## 1 INTRODUCTION

Many *Bartonella* species, which are facultative intracellular, fastidious, Gram‐negative alpha‐proteobacteria, have been described so far (Okaro et al., 2017). These bacteria are highly adapted to one or more mammalian hosts, infecting erythrocytes and endothelial cells, establishing a long‐term silent infection in mammalian reservoirs, through escaping the immune response (Harms & Dehio, 2012). The spread of *Bartonella* spp. infection among mammalians occurs mainly via bloodsucking arthropod vectors, particularly fleas (Kosoy et al., 2012), disseminating the bacteria within specific reservoir communities and between different reservoirs. Currently, out of 40 *Bartonella* species/subspecies, at least 17 are associated with an expanding spectrum of clinical signs in humans and animals (Breitschwerdt, 2014; Chomel & Kasten, 2010; Harms & Dehio, 2012; Okaro et al., 2017).

More than 60% of human pathogens are of animal origin with the majority coming from wildlife (Böhm et al., 2007). National or regional parks and protected areas offer habitats for such a diverse wild fauna, thus acting as potential reservoirs for many human and domestic animal pathogens including *Bartonella* spp. (Ambrogi et al., 2019; Millán et al., 2016). In Southern Italy, wild mammal populations in the National (the Cilento and Vallo di Diano) and Regional (Partenio and Monti Picentini) Parks have increased in recent decades. For instance, the Eurasian wolf (*Canis lupus*) population, close to extinction due to human activities until 1970s, has rebounded, with an estimated 1,500–1,800 individuals thanks to conservation policies and to an increase in its main prey species (Galaverni et al., 2015). In addition, red fox (*Vulpes vulpes*) is a significant free‐ranging carnivore in these areas. *Bartonella* infection in foxes has been described worldwide (Bai et al., 2016; Chomel et al., 2012; Fleischman et al., 2015; Gerrikagoitia et al., 2012; Henn, Chomel, et al., 2009; Hodžić et al., 2018; Kosoy & Goodrich, 2019; López‐Pérez et al., 2017; Marciano et al., 2016; Millán et al., 2016; Schaefer et al., 2011; Víchová et al., 2018), whereas it has rarely been reported in wolves from Spain (Gerrikagoitia et al., 2012). Since both wolf and red fox are sympatric with dogs especially feral and rural ones, they may play an important role in the *Bartonella* ecology (Bateman & Fleming, 2012; Gehrt et al., 2013). Finally, a number of badgers, hedgehogs, martens and roe deers are registered in the area, all of them shown to be susceptible to *Bartonella* spp. infection (Bitam et al., 2009, 2012; Dehio et al., 2001; Gerrikagoitia et al., 2012; Harms et al., 2017; Sato et al., 2012). In Italy, *B. bovis* and *B. chomelii* are the only species reported in wildlife, from deer ticks (Ebani et al., 2015). Notably, data on wild canid infections are lacking, although *B. vinsonii* subsp. *berkhoffii* and the uncultured *Bartonella* sp. strain HMD later shown to be *Candidatus* B. merieuxii have been detected in hunting and rural dogs in southern Italy (Chomel et al., 2012; Diniz et al., 2009). It is worth mentioning that *B. vinsonii* subsp. *berkhoffii* genotype III can be highly pathogenic for dogs (Shelnutt et al., 2017).

To gain insight for potential biologic threats for wildlife and for humans and their domestic animals within the One Health approach (Breitschwerdt, 2014), this study aimed at investigating the occurrence of *Bartonella* species in different free‐ranging wildlife in South Italy.

## 2 MATERIALS AND METHODS

### 2.1 Study area

**FIGURE 1:**
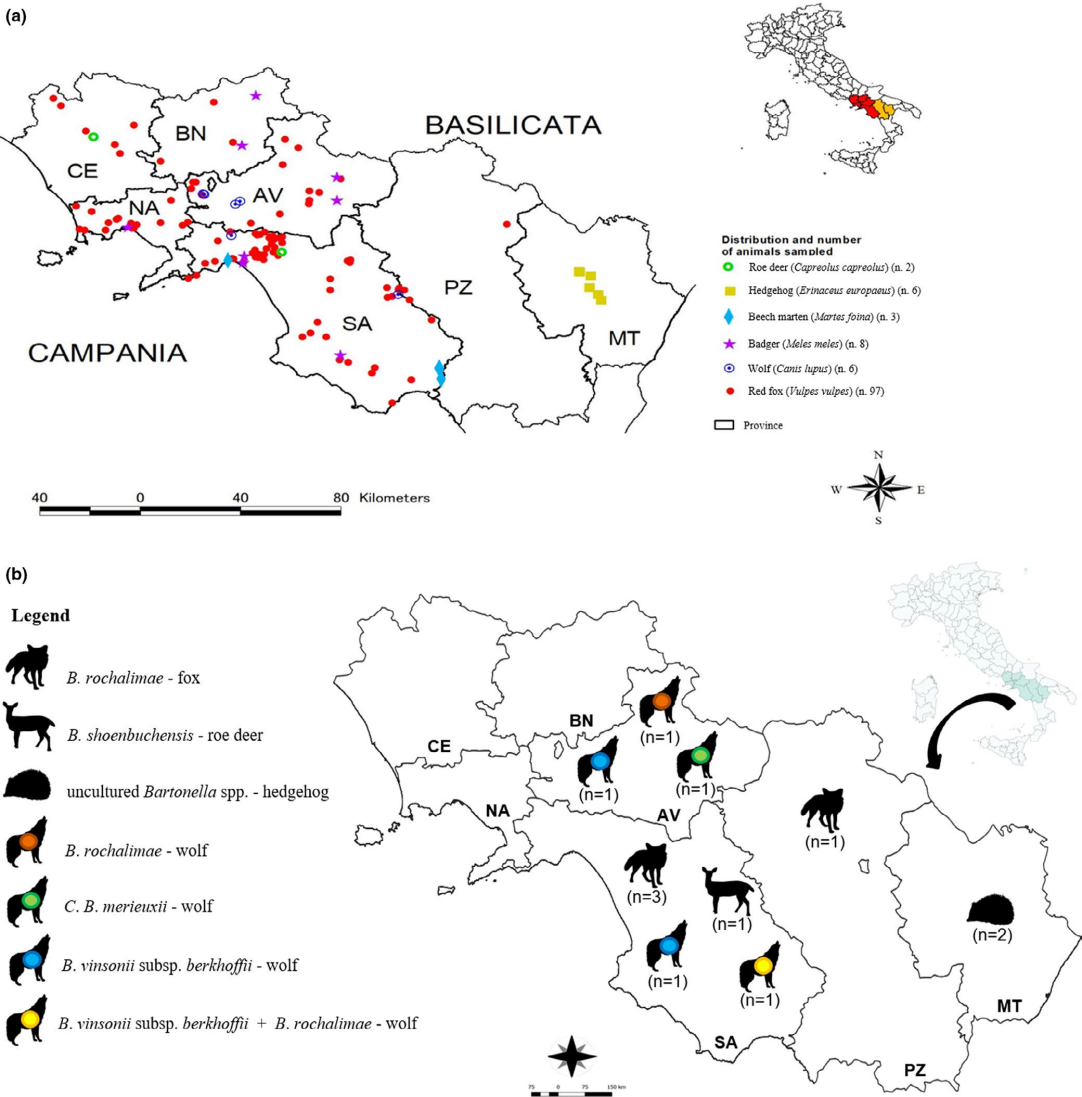
(a) Sampling sites, number and distribution of wolves, red foxes, badgers, martens, roe deer and hedgehogs according to the administrative regional and provincial boundaries of the study area: Campania region (AV ‐ Avellino, BN ‐ Benevento, CE ‐ Caserta, NA ‐ Naples, SA – Salerno); Basilicata region (PZ ‐ Potenza, MT ‐ Matera). (b) Number and distribution of wildlife positive to different *Bartonella* species according to the administrative regional and provincial boundaries of the study area. Campania region (AV ‐ Avellino, BN ‐ Benevento, CE ‐ Caserta, NA ‐ Naples, SA – Salerno); Basilicata region (PZ ‐ Potenza, MT ‐ Matera)

The survey included seven provinces of the Campania and Basilicata administrative regions located in the southwest of the Italian peninsula, with the Tyrrhenian Sea to the west and the Campania‐Lucania Apennines and the Ionian Sea to the south (Figure 1a). The area includes both densely (Caserta, 41°1′N, 14°19′E; Napoli, 40°50′N, 14°15′E; Salerno, 40°41′N, 14°47′E) and low populated human habitats including Partenio (40°57′N, 14°40′E) and Monti Picentini (40°43′N, 14°56′E) Regional Parks, and the Cilento and Vallo di Diano National Park (40°18′N, 15°23′E) (the second‐largest in Italy), respectively. Altitude ranges from sea level to close to 1,900 metres. The territory covers complex ecosystems and habitats allowing the presence of such diverse fauna (Guglietta et al., 2015).

### 2.2 Study animals

Spleen samples from 122 carcasses including 97 (79.5%) red foxes (*Vulpes vulpes*), 8 (6.6%) badgers (*Meles meles*), 6 (4.9%) Eurasian wolves (*Canis lupus*), 6 (4.9%) hedgehogs (*Erinaceus europaeus*), 3 (2.5%) beech martens (*Martes foina*), and 2 (1.6%) roe deer (*Capreolus capreolus*) were collected between 2016 and 2019 in seven provinces of both Campania and Basilicata administrative regions (Table 1, Figure1a). According to the administrative boundaries (i.e. provinces) of the study area, the distribution of the animal samples was depicted using the arcgis software (version 10.3; ESRI, Redlands, CA, USA). Categorical age, sex and location data were recorded. The survey did not involve any direct manipulation of animals and relied entirely on the collection of road‐killed animals, except for the red fox samples obtained during the official hunting season from local hunters. After necropsy performed at the Istituto Zooprofilattico Sperimentale del Mezzogiorno–Portici (IZSM) the spleen samples were sent to the Veterinary Medicine Department of the University of Bari Aldo Moro, Italy. All samples were stored at −20°C until use.

**TABLE 1:** Characteristics and *Bartonella* spp. infection rates of wild animals from Italy

	*N* ^a^	Gender	Age^b^	Weight^c^	*Bartonella* spp.
	122	*n* (%) F/M	*n* (%) A/S	kg	*n* (%) F/M
*Carnivora*					
(*Canis lupus*) Eurasian Wolf	6	1 (17)/5(83)	6 (100) adults	39.2	5 (83.3) 1/4
(*Vulpes vulpes*) Red Fox	97	43 (44.3)/54(5.7)	80 (82.5)/17 (17.5)	4.90	4 (4.12) 3/1
(*Meles meles*) European badger	8	3 (37.5)/5(62.5)	8 (100) adults	11	0
(*Martes foina*) Beech marten	3	2 (66.6)/ 1 (33.3)	3 (100) adults	1.5	0
*Cervidae*					
(*Capreolus capreolus*) Roe deer	2	1/1 (50)	2 (100) adults	37.5	(50) 1 F
*Erinaceomorpha*					
(*Erinaceus europaeus)* Hedgehog	6	3/3 (50)	3/3 (50)	n.d	2 (33.3) 1/1

^a^ Number of sampled animals.

^b^ A, adult = >1 year; SA, sub‐adult = <1 year.

^c^ Mean weight.

### 2.3 Molecular procedures

All the spleen samples were homogenized in Minimal Essential Medium (MEM, 50 mg/ml). DNA was extracted from 200 µl of homogenate using the DNeasy Tissue Kit (Qiagen, Milan, Italy) according to the manufacturer’s instructions. DNA was eluted in 100 μl of AE buffer and stored at −20°C till testing. For quality assurance, a *Bartonella*‐free spleen sample as an extraction negative control was used. DNA was carefully quantified using the fluorometric Qubit^®^ dsDNA HS (High Sensitivity) Assay kit, and the extracts were diluted at the final concentration of 2.5 ng/μ. Two microl were used for each qPCR and cPCR assays. Samples were screened using a *Bartonella genus*‐specific quantitative real‐time PCR (qPCR) assay targeting the transfer‐mRNA *ssr*A (*ssr*A) gene as previously described (Diaz et al., 2012) (Table 2). qPCR amplification was conducted in Multiplate PCR plates (Bio_Rad^™^, Milan, Italy) using a CFX96 Touch Real‐Time PCR Detection System (Bio_Rad^™^, Milan, Italy). DNS load for each sample was calculated by using the standard curve generated with different 10‐fold dilutions (1.0 × 10^0^ to 1.0 × 10^9^ copies per 10 μl) of the plasmid DNA encoding a 300‐bp *B. henselae ssr*A gene fragment. The *ssrA* qPCR positive samples were further tested using additional conventional PCR (cPCR) assays that amplify the citrate synthase (*glt*A), *ssr*A, and RNA polymerase beta subunit‐encoding (*rpo*B) housekeeping genes, and 16S–23S ITS target fragment (Table 2) (Birtles & Raoult, 1996; Diaz et al., 2012; Diniz et al., 2007; Oksi et al., 2013). PCR products displaying high intensity of band with expected sizes were purified using the NEB Exo‐SAP PCR purification kit (New England Biolabs, Inc., Ipswich, MA, USA) and sequenced for speciation and phylogenetic analysis by Eurofins Genomics (Vimodrone, Italy). Reference strains *B. clarridgeiae* (MH348146), *B. henselae* (MH350809), *B. rochalimae* (MK780191) and *B. vinsonii* subsp. *berkhoffii* (MK773857) were used as positive controls for each cPCR. Nucleotide sequences were compared with GenBank entries by Basic Local Alignment Search Tool (BLAST) (https://blast.ncbi.nlm.nih.gov/Blast.cgi). Chromatogram evaluation, primer deletion and sequence alignment were performed using the geneious ^®^ 10.3.1 software package (Biomatters Ltd., Auckland, New Zealand). The Clustal W program was used to align each sequence and compare homologous gene/target to identify genetic variants. For phylogenetic analyses, partial ITS, *glt*A, *rpo*B and *ssr*A new sequences and those from representative known *Bartonella* isolates were analysed with mega‐x v. 10.0.5 software (Kumar et al., 2018). Phylogeny inference was calculated using ‘find best DNA/protein model’ tool from MEGAX. Maximum likelihood method with Tamura Nei 3‐parameter substitution model, a proportion of invariable sites and a gamma distribution of rate variation across sites was applied supplying statistical support with subsampling over 1,000 replicates.

**TABLE 2:** Primers used for quantitative and conventional PCR

Target	Primer Name	Sequence	bp	References
*ssr*A	*ssr*A_F	GCTATGGTAATAAATGGACAATGAAATAA	300	(Diaz et al., 2012)
	*ssr*A_R	GCTTCTGTTGCCAGGTG		
	FAM‐labelled probe	ACCCCGCTTAAACCTGCGACG		
ITS	325_F	CTTCAGATGATGATCCCAAGCCTTYTGGCG	673	(Diniz et al., 2007)
	1100_R	GAACCGACGACCCCCTGCTTGCAAAGCA		
*rpo*B	prAPT0244_F	GATGTGCATCCTACGCATTATGG	406	(Oksi et al., 2013)
	prAPT0245_R	AATGGTGCCTCAGCACG TATAAG		
*glt*A	443_F	GCTATGTCTGCATTCTATCA	340	(Birtles & Raoult, 1996)
	781_R	CCACCATGAGCTGGTCCCC		

### 2.4 Statistical analysis

An animal was considered *Bartonella* spp. infected if it was positive in the qPCR. Exact binomial 95% confidence intervals (CIs) were established for proportions. Proportion differences were tested for statistical significance using the Fisher’s exact test/or Chi square, where appropriated. The statistical significance threshold for both tails test was set at *p* ≥ .05. All statistics were performed on winepi software (http://winepi.net/; October 2020).

## 3 RESULTS

The concentration mean from the DNA spleen samples extracted was 10.18 ng/μl (ranging from 4.0 to 14.1 ng/L; standard deviation [*SD*], 2.92). The efficiency mean of qPCR assays was *E* 94.84%, slope 3.45, *r* ^2^ 0.997, intercept: 41.50.

Molecular screening with *ssr*A qPCR assay detected *Bartonella* spp. DNA in 12 (9.84%; 95% CI: 4.55%–15.12%) out of 122 spleen samples from four different wildlife species (wolves, foxes, hedgehogs and roe deer) but none from either the badgers or martens (Table 3). *Bartonella* DNA load ranging between 4.84 × 10^0^ and 2.59 × 10^4^ (2.27 × 10^3^ mean, 5.15 × 10^1^ median) DNA copies per μl. The frequencies and the spatial distribution of the detected *Bartonella* species are shown in Tables 1 and 3 and Figure 1.

**TABLE 3:** *Bartonella* species molecularly detected in wildlife spleen samples from the provinces of Campania and Basilicata regions, southern Italy

Host Species (Common name)	Sample ID^a^	*Bartonella* species^b^	Loci ID^c^	Similarities (% reference strain)	Accession number
*Canis lupus* (Eurasian wolf)	Cl_1 and 2	*Bvb*III	ITS	99.42% AF143446	MW042398
			*ssr*A	100% CP003124	MW042399
*Canis lupus* (Eurasian wolf)	Cl_3a	*Bvb*III	ITS	99.42% AF143446	n.s
	Cl_3b	*Br*	*glt*A	100% CP019780	n.s.
			*rpo*B		n.s.
*Canis lupus* (Eurasian wolf)	Cl_4	*Br*	ITS	100% CP019780	MW042394 *
			*glt*A		MW042395 *
			*rpo*B		MW042396 *
			*ssr*A	99.19% CP019780	MW042397*
*Canis lupus* (Eurasian wolf)	Cl_5	*CBm*	*rpo*B	99.44% EF592104	MW042400
			*ssr*A	99.57% MK780190	MW042401
*Vulpes vulpes* (Red fox)	Vv_1–4	*Br*	ITS	100% CP019780	n.s
			*glt*A		n.s.
			*rpo*B		n.s.
			*ssr*A		n.s.
*Capreolus capreolus* (Roe deer)	Cc_1	*Bs*	ITS	99.70% CP019789	MW042391
			*rpo*B	99.46% CP019789	MW042392
			*ssr*A	100% CP019789	MW042393
*Erinaceus europaeus* (European hedgehog	Ee_1 and 2	*UB_Ee*	ITS	85.57% AF312497	MW042389
			*ssr*A	95.97% MK298164	MW042390

^a^ *Cl: Canis lupus; Vv: Vulpes vulpes; Cc: Capreolus capreolus, Ee: Erinaceus europeus. Cl*‐1: wolf 11; *Cl*‐2: wolf 30; *Cl*‐3a and *Cl*‐3b: wolf 17; *Cl*‐4: wolf 15; *Cl*‐5: wolf 16; *Vv*1: red fox 6; *Vv*2: red fox 10; *Vv*3: red fox l 44; *Vv*4: red fox 111; *Cc‐*1: roe deer 25; *Ee*‐1: hedgehog 31; *Ee*‐2: hedgehog 33.

^b^ *Bvb*III: *B. vinsonii* subsp. *berkhoffii* type III; *Br*: *B. rochalimae*; *C*Bm: *Candidatus* Bartonella merieuxii; *Bs*: *B. schoenbuchensis*; UB_Ee: Uncultured *Bartonella* spp. from Ee.

^c^ Obtained and sequenced *loci* (cPCR products).

* Asterisks denote the nucleotide sequences identical to strains retrieved from red foxes.

**FIGURE 2:**
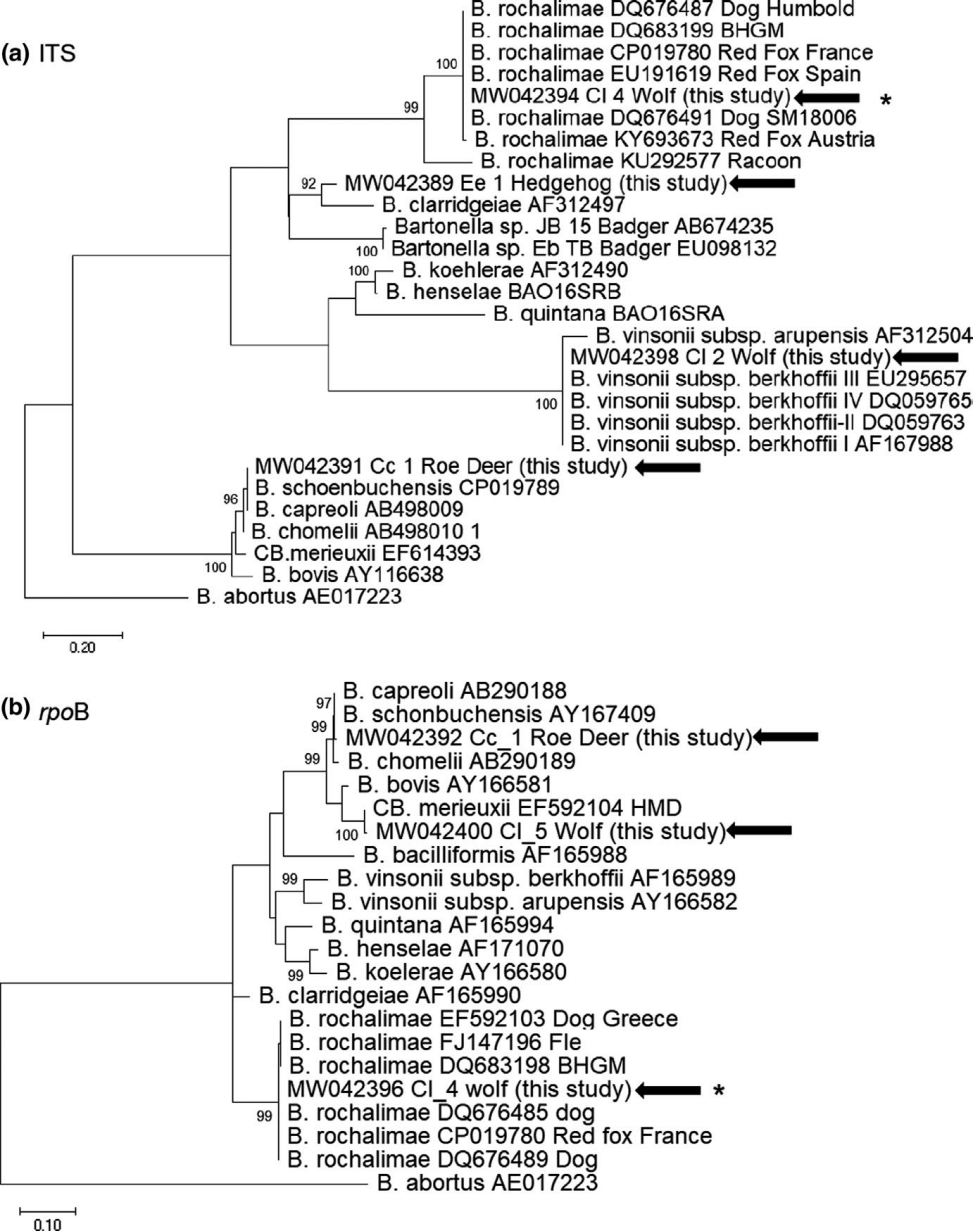
Phylogenetic trees displaying the diversity of *Bartonella* species detected in wild mammals from Italy. The phylogenetic trees for partial ITS (600 bp) (panel a) and *rpo*B (357 bp) (panel b) sequences from representative known *Bartonella* isolates were generated by using MEGA‐X v. 10.0.5 software. Maximum likelihood method with Tamura Nei 3‐parameter substitution model, a proportion of invariable sites and a gamma distribution of rate variation across sites was applied supplying statistical support with subsampling over 1,000 replicates. GenBank accession numbers are provided for reference isolates with the sequence from *Brucella abortus* (AE017223) used as outgroup. The representative sequences generated in the present study are marked with arrows. Asterisks denote the nucleotide sequences identical to strains retrieved from red foxes. Scale bars indicate nucleotide substitutions per site

**FIGURE 3:**
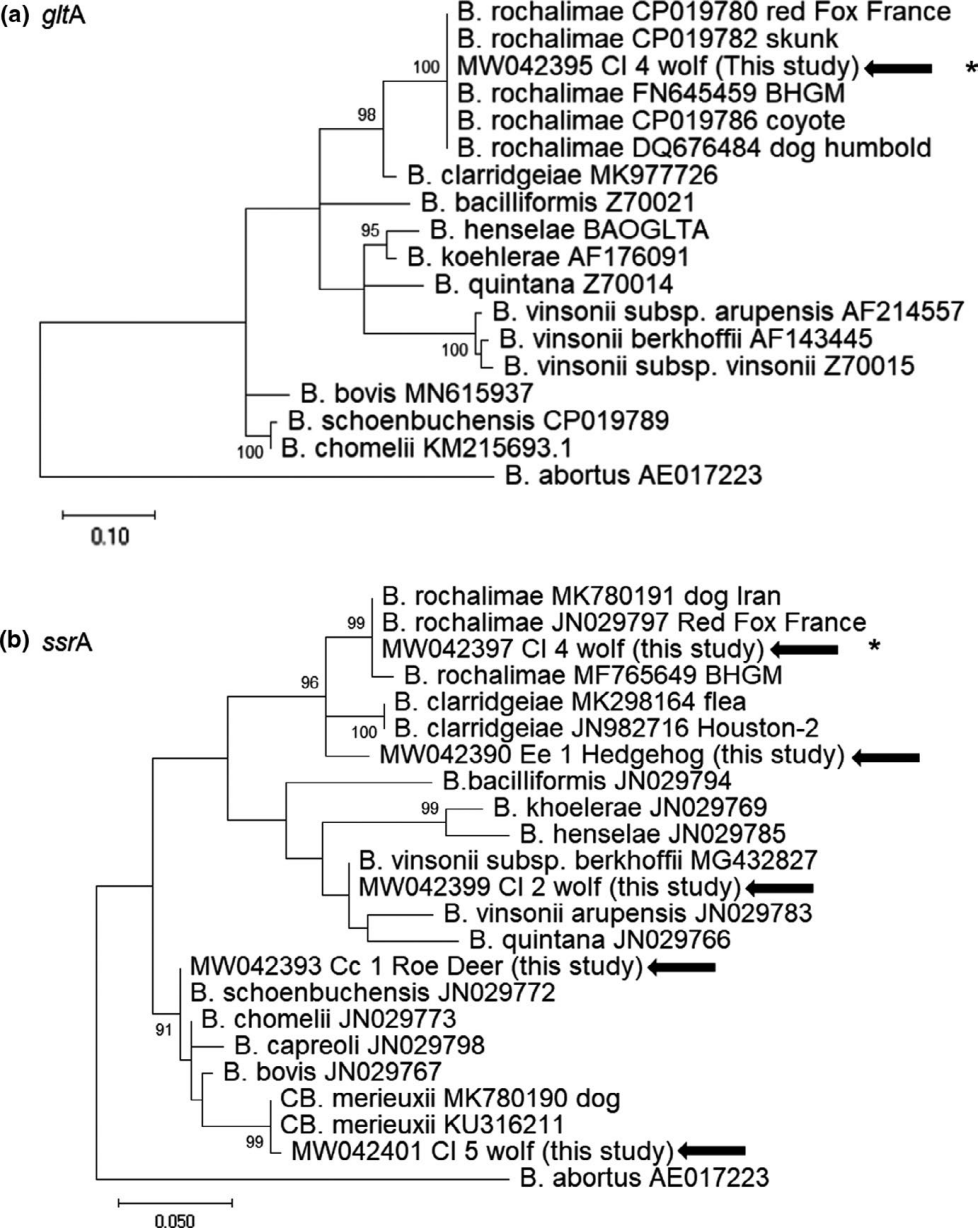
Phylogenetic trees displaying the diversity of *Bartonella* species detected in wild mammals from Italy. The phylogenetic trees for partial *glt*A (290 bp) (panel a), and *ssr*A (254 bp) (panel b) sequences from representative known *Bartonella* isolates were generated by using mega‐x v. 10.0.5 software. Maximum likelihood method with Tamura Nei 3‐parameter substitution model, a proportion of invariable sites and a gamma distribution of rate variation across sites was applied supplying statistical support with subsampling over 1,000 replicates. GenBank accession numbers are provided for reference isolates with the sequence from *Brucella abortus* (AE017223) used as outgroup. The representative sequences generated in the present study are marked with arrows. Asterisks denote the nucleotide sequences identical to strains retrieved from red foxes. Scale bars indicate nucleotide substitutions per site

Furthermore, out of 12 qPCR positive samples, 6, 8, 11, and 11 were positive through cPCR assays targeting the *gltA*, the *rpo*B, the *ss*rA and ITS *loci*, respectively (Table 3). In details, *glt*A sequences were retrieved from 2 wolves (#Cl_3b and Cl_4) and 4 foxes (#Vv‐1 to 4). The *rpoB* sequences were detected from one roe deer (#Cc_1), 4 foxes (#Vv‐1 to 4) and 3 wolves (#Cl_3b, Cl_4 and #Cl_5). The *ssr*A and ITS sequences were identified from all the hosts except from the #Cl_3 and #Cl_5 wolves, respectively (Table 3 and Figures 2 and 3).

Five (83.3%; 95% CI: 51%–100.00%) of 6 carcasses of Eurasian wolf were positive for *Bartonella* DNA (Tables 1 and 3, Figure 1b and Figures 2 and 3). *B. rochalimae* DNA sequence was detected in 1 (#Cl_4) of these five wolves (Table 3). Two (#Cl_1 and Cl_2) other wolves (2/5) were infected with *B. vinsonii* subsp. *berkhoffii* type III. One (#Cl_5) wolf harboured DNA sequences similar to *Candidatus* Bartonella merieuxii clones (Table 3). Lastly, one (#Cl_3) wolf (1/5) was co‐infected with *B. vinsonii* subsp. *berkhoffii* and *B. rochalimae* (Table 3, Figures 2 and 3). The *rpo*B sequence of the *Candidatus* B. merieuxii from Italian wolf was 99.22% similar to HMD clone detected in rural dogs in South Italy (Diniz et al., 2009) and to F040 clone (Chomel et al., 2012) from Iraqi jackal. The *ssr*A sequence was 99.57% similar to the Iran‐GT‐3b clone from an Iranian dog and to clone Ca‐1 detected in a jackal from Israel, respectively (Table 3) (Greco, Sazmand, et al., 2019; Marciano et al., 2016). No significant differences were recorded for locality (*p* = 1) or gender (*p* = .96).

Most of the red foxes were adults (*n* = 80; 82.5%) and males (*n* = 54; 56.7%) (Table 1). The geographic origin of the red fox carcasses is displayed in Figure 1. Four (i.e. Vv_1 to 4) spleen samples (4.12%; 95% CI: 0.17%–8.08%) were positive for *Bartonella* DNA sequences, all of them being 100% identical to each other and to those ones from 2 (#Cl_3b and Cl_4) wolves (Table 3, Figures 2 and 3). By phylogenetic analyses, *glt*A and *rpo*B sequences were 99.8 to 100% identical to *B. rochalimae* strains isolated from red foxes in Europe (Henn, Chomel, et al., 2009; Hodžić et al., 2018; Millán et al., 2016) (Tables 1 and 3, Figures 2 and 3). The ITS sequences confirmed 100% identity with *B. rochalimae* strains previously detected from foxes or wolves (Henn, Chomel, et al., 2009; Hodžić et al., 2018; Millán et al., 2016) but displaying small differences (2 to 5 bp) when compared with strains from human and their fleas or rural dogs (Diniz et al., 2009; Eremeeva et al., 2007; Henn, Chomel, et al., 2009; López‐Pérez et al., 2017; Parola et al., 2002) (Tables 1 and 3, Figures 2 and 3).

There was no significant statistical difference for gender (*p* = .32) or locality (*p* > .5). After comparing *Bartonella* spp. distributions between carnivorous species, wolves were statistically more likely to be infected than foxes (χ^2^ = 44.46, *df* = 1, *p* < .0001).

Out of the two adult roe deer, the female tested positive for *Bartonella* DNA. Sequence analysis of 3 targets (ITS, *rpo*B, *ssr*A) allowed the identification of *B. schoenbuchensis*. The ITS, *rpo*B, *ssr*A sequences displayed the highest similarity, 99.70%, 99.46% and 100%, with the *B. schoenbuchensis* R1 strain isolated from a blood sample of a roe deer from Germany (Table 3, Figures 2 and 3) (Dehio et al., 2001). Animals were equally distributed for age and gender (Table 1).

Out of the six hedgehog carcasses, all of them collected along the Matera province, Basilicata region, from the Centro Recupero Animali Selvatici (CRAS), 2 spleen samples (33.33%; 95% CI: 0.00%–71.05%) were positive for *Bartonella* DNA sequences (*ssr*A and ITS) 100% identical to each other (Tables 1 and 3). The *ssr*A sequence displayed highest similarities, 95.97% and 95.51, to some *B. clarridgeiae*‐like BFP clones detected from fleas from cats (Abdullah et al., 2019) and to *B. clarridgeiae* Houston‐2 strain (Table 3, Figures 2 and 3). Further, after comparing the ITS sequence to those of the *Bartonella* types/reference strains, the results indicated that these hedgehog isolates did not belong to any known *Bartonella* species, with DNA similarity values to *B. clarridgeiae* less than 85.57% (Table 3). However, by phylogenetic analyses based on both ITS and *ssr*A sequences, these hedgehog *Bartonella* clones grouped in the same clade including *B. clarridgeiae*, *B. rochalimae,* strain JB15 isolated from a Japanese badger and an uncultured T8 clone detected from an European badger (Figures 2 and 3) (García‐Esteban et al., 2008; Harms et al., 2017; Sato et al., 2012).

### 3.1 Nucleotide sequence accession numbers

The novel unique sequences obtained in the present study have been deposited in GenBank^®^ database and they are available under the following accession numbers: MW042398 (*B. vinsonii* subsp. *berkhoffii* type III, 16S–23S rRNA target), MW042399 (*B. vinsonii* subsp. *berkhoffii* type III, *ssr*A gene), MW042394* (*B. rochalimae,* 16S‐23S rRNA), MW042395* (*B. rochalimae, glt*A gene), MW042396* (*B. rochalimae, rpo*B gene), MW042397* (*B. rochalimae, ssr*A gene), MW042400 (*Candidatus* Bartonella merieuxii, *rpo*B gene), MW042401 (*Candidatus* Bartonella merieuxii, *ssr*A gene), MW042391 (*B. schoenbuchensis,* 16S–23S rRNA), MW042392 (*B. schoenbuchensis, rpo*B gene), MW042393 (*B. schoenbuchensis, ssr*A gene), MW042389 (Uncultured *Bartonella* spp., 16S–23S rRNA), MW042390 (Uncultured *Bartonella* spp., *ssr*A gene) (Table 3, Figures 2 and 3). Asterisks denote the nucleotide sequences identical to strains retrieved from red foxes.

## 4 DISCUSSION

A wide range of *Bartonella* species, including several zoonotic ones, was detected among four free‐ranging mammalian species (red foxes, wolves, hedgehogs and roe deer) from Nature preserve parks in southern Italy. *Bartonella rochalimae* was the only species detected in red foxes and was one of the three *Bartonella* species detected in wolves. *Candidatus* B. merieuxii and *B. vinsonii* subsp. *berkhoffii* were identified for the first time in wolves. It is also the first report of the presence of *B. schoenbuchensis* in a roe deer in Italy. Furthermore, based on ITS and *ssr*A phylogenetic analyse, a new uncultured *Bartonella* clone, segregating within the same clade including *B. clarridgeiae* and *B. rochalimae,* was identified in European hedgehogs. Although the small size of the sample tested and the lacking of checking for PCR inhibitors in DNA samples may prevent from any conclusive result, *Bartonella* spp. were not detected in badgers and beech martens, as previously reported in Spain (Márquez et al., 2009; Millán et al., 2016).

*Bartonella* infections in wild and domestic canids have been reported worldwide (Bai et al., 2016; Chomel et al., 2012; Fleischman et al., 2015; Gerrikagoitia et al., 2012; Greco, Sazmand, et al., 2019; Henn, Chomel, et al., 2009; Hodžić et al., 2018; Kosoy & Goodrich, 2019; López‐Pérez et al., 2017; Marciano et al., 2016; Millán et al., 2016; Schaefer et al., 2011; Víchová et al., 2018). However, in Italy, only one seroprevalence survey in domestic dogs had reported a 6% prevalence for *B. henselae* infection in owned dogs in the Tuscany region (Ebani, Nardoni, et al., 2015). In addition, DNAs of *B. vinsonii* subsp. *berkhoffii* type III and of the uncultured *Bartonella* spp. strain HMD later shown to be *Candidatus* B. merieuxii were detected in rural and hunting dogs from the south and the centre of the country (Chomel et al., 2012; Diniz et al., 2009; Ebani, Nardoni, et al., 2015). Up to date, no information was available on *Bartonella* infection in wild canids in Italy. DNA sequences of different *Bartonella* spp. were detected in wild canids with occurrence being much higher in Eurasian wolves (>80%) than in red foxes (<5%), thus identifying this carnivore as significant reservoir for *Bartonella* spp. infection in southern Italy. Though the occurrence observed in the present study is probably biased because of the small wolf and fox sample sizes, it may be affected by the different behaviours of the two canids. Indeed, the close physical contact between group members of social canids such as wolves, rather than the lonely foxes may have enhanced the likelihood of transmission of pathogens and vectors between wolves (Delamater et al., 2019). Unfortunately, ectoparasite infestation could not be investigated as the arthropods, in particular the fleas, promptly abandon the carcasses soon after the animal death. Thus, future studies need to evaluate their role on *Bartonella* spp. infections in these geographical locations. Moreover, no statistically significant relationship was observed between *Bartonella* spp. infection and gender, age, or localities.

A *B. rochalimae* strain, 99.8% to 100% identical to the strains isolated from red foxes in France, Austria and Spain was circulating among both wolves (33.3%) and red foxes (4.12%) from the Cilento and Vallo di Diano National Park, supporting the evidence that the two animal species share ecosystems and pathogens (Henn, Chomel, et al., 2009; Hodžić et al., 2018; Millán et al., 2016). The discordant values of *B*. *rochalimae* occurrence observed in the two animal species overlapped previous studies in northern Spain, where prevalence of 33.3 % and 1.6% had been reported in wolves and foxes, respectively (Gerrikagoitia et al., 2012). The zoonotic *B. rochalimae* species has been recorded worldwide in coyotes, wolves, island foxes, grey foxes, red foxes, raccoons, skunk and domestic dogs (Fleischman et al., 2015; Gerrikagoitia et al., 2012; Greco, Sazmand, et al., 2019; Henn, Chomel, et al., 2009; López‐Pérez et al., 2017; Marciano et al., 2016; Millán et al., 2016; Schaefer et al., 2011). The present study revealed the circulation of such a zoonotic agent within wild canids in regional and national parks in Southern Apennines, thus expanding knowledge about the spatial distribution of *B. rochalimae* in wild in Italy.

Further, *B. vinsonii* subsp. *berkhoffii* type III was detected in 3 out 5 wolves one of which was co‐infected with *B. rochalimae*. Previous molecular studies had already described *B. vinsonii* subsp. *Berkhoffii* type III in rural (3.3%) and hunting dogs (20%) in Italy, but to the best of our knowledge this represents the first detection in the Eurasian wolf (Diniz et al., 2009; Ebani, Nardoni, et al., 2015). Both *B. vinsonii* subsp. *berkhoffii* and *B. rochalimae* have been associated with clinical signs, mainly fever, endocarditis and myocarditis, in dogs and humans (Eremeeva et al., 2007; Henn, Gabriel, et al., 2009; Roux et al., 2000; Shelnutt et al., 2017). Our study confirms wild carnivores as natural reservoirs of both *B. rochalimae* and *B. vinsonii* subsp. *berkhoffii*, thus revealing a real risk for humans and dogs.

Lastly, one wolf carcass recovered in the Regional Partenio Park in Avellino province was infected with the recently proposed species *Candidatus* B. merieuxii. By sequence analyses based on *rpo*B and *ssr*A the *Bartonella* detected strain matched with HMD and Iran‐GT‐3b clones detected in rural dogs from South Italy and Iran (Diniz et al., 2009; Greco, Sazmand, et al., 2019), and with the F040 and Ca‐1 clones from Iraqi and Israel jackals (Chomel et al., 2012; Marciano et al., 2016), respectively. The results of this study show that *C*. B. merieuxii is also infecting in Eurasian wolf, thus expanding the host range of this *Bartonella* species.

A female roe deer from the Regional Park of Monti Picentini in the Salerno province was infected with a *B. schoenbuchensis* strain matching with the R1 strain isolated from a roe deer blood sample from Germany (Dehio et al., 2001). The homology with the reference strain (GenBank accession number CP019789) for *rpo*B, ITS and *ssr*A ranged from 99.46% to 100%. *Bartonella schoenbuchensis* is endemic in roe deer and their ticks (70%) in south Germany (Dehio et al., 2001). In Italy, *B. chomelii* and *B. bovis* were detected in high numbers in tick pools from roe deer in Tuscany, but to our best knowledge, this is the first detection of *B. schoenbuchensis* in the country*. *Although the pathogenic role of this *Bartonella* species in ruminants has not been fully determined, its zoonotic potential should be carefully investigated, as its active arthropod vector *Liptotena cervi* (Diptera: *Hippoboscidae*), recently detected in Italy, can bite humans (Bezerra‐Santos & Otranto, 2020; de Bruin et al., 2015; Salvetti et al., 2020; Szewczyk et al., 2017).

*Bartonella* infection in European hedgehogs had not been investigated prior to the present study. Several zoonotic *Bartonella* species (*B. elizabethae*, *B. tribocorum*, or *B. clarridgeiae*) have been detected in African hedgehogs (*Atelerix algirus*) (26%) and their fleas (*Archeopylla erinacei*) (0.045%) in Algeria (Bitam et al., 2009; Gehrt et al., 2013). An uncultured *Bartonell*a spp. was detected in Southern white‐breasted hedgehogs (33.3%) in Israel (Marciano et al., 2016). In the present study, a new *Bartonella* clone was detected in the spleen of two (33.3%) European hedgehogs from peri‐urban areas in Matera province (Basilicata region). By phylogenetic analyses of the ITS and *ssr*A sequences, the new detected *Bartonella* clone grouped in the same *clade* including *B. clarridgeiae* and *B. rochalimae* (García‐Esteban et al., 2008; Harms et al., 2017; Sato et al., 2012). Furthermore, *ssr*A sequences (of the new uncultured *Bartonella* clone) revealed similarity (95.97%) to some *B. clarridgeiae* strains detected in cat fleas from the United Kingdom (Abdullah et al., 2019). Based on previous studies detecting *B. clarridgeiae* in flea infested bacteraemic cats (1.21%, 95% CI: 0.03–2.39) with outdoor life style in south Italy (Greco, Brianti, et al., 2019; Otranto et al., 2017) further research should be conducted to investigate whether the *Bartonella* clone described in hedgehogs could be transmitted to cats through common vectors. These findings point to a cautious management of such animals hosting strains phylogenetically related to the zoonotic *B. clarridgeiae* species that is known to be a minor agent of CSD with cats and their fleas as reservoirs (Clarridge et al., 1995; Kim et al., 2009).

In conclusion, our findings stress the importance of wildlife disease surveillance, mainly for wildlife protection and as a useful and complementary component of human and domestic animal disease surveillance. Noteworthy, these findings underline the risk of exposure to *Bartonella* spp. infections in nature lovers, orienteering/trekking competitors, hunters, wildlife rangers and local residents during outdoor activities along with their domestic animals, as they can easily encounter wildlife animals, arthropods and, eventually, be in contact with the pathogens they transmit.

## CONFLICT OF INTEREST

The authors declare that they have no conflict of interest.

## ETHICAL APPROVAL

The study was conducted under the frame of a wildlife health‐monitoring plan authorized by the Regione Campania (Animal Ethics Committee, Decreto Dirigenziale 96 no. 210 ‐ Piano B7 DPAR 2018) in order to assess the health status of domestic and wild free‐ranging animals. Procedures according to the Italian Ministry of Health guidelines were strictly applied.

## Data Availability

The data that support the findings of this study are available from the corresponding author upon reasonable request.
